# Enhancing the Reliability of AD936x-Based SDRs for Aerospace Applications via Active Register Scrubbing and Autonomous Fault Recovery

**DOI:** 10.3390/s25216801

**Published:** 2025-11-06

**Authors:** Jinyang Wang, Zhugang Wang, Li Zhou

**Affiliations:** 1 National Space Science Center, Chinese Academy of Sciences, Beijing 100190, China; wangjinyang21@mails.ucas.edu.cn (J.W.); zhouli@nssc.ac.cn (L.Z.); 2 School of Electromagnetic Field and Microwave Technology, University of Chinese Academy of Sciences, Beijing 100049, China

**Keywords:** Software-Defined Radio (SDR), fault tolerance, Fault Detection, Isolation, Recovery (FDIR), Single Event Upset (SEU), aerospace reliability, register scrubbing

## Abstract

Single Event Upsets (SEUs) in Commercial Off-The-Shelf (COTS) Software-Defined Radios (SDRs) are frequent in a erospace applications, especially in GEO (Geostationary Orbit) orbit during severe solar activity, and can lead to unexpected register corruption and communication failures. This work presents a purely software-based Fault Detection, Isolation, and Recovery (FDIR) framework tailored for the AD936x RF agile transceiver, requiring no hardware modifications. The proposed method classifies all device registers into four impact categories and applies dedicated scrubbing strategies—standard refresh, masked refresh, procedural refresh, and forced refresh—combined with real-time register health monitoring and adaptive recovery actions. Fault injection experiments comprising 10,000 diverse test cases achieved 100% fault coverage for the tested scenarios, with an average recovery time of 0.75 s for typical SEUs and a guaranteed worst-case recovery under 4.4 s for critical failures, while maintaining a CPU load below 1.3%. The approach ensures continuous SDR operation under SEU events and offers a scalable, lightweight, and cost-effective reliability enhancement for CubeSats and other resource-constrained aerospace platforms.

## 1. Introduction

Software-Defined Radio (SDR) technology offers unprecedented flexibility for modern aerospace communication systems, enabling in situ reconfiguration and multi-band operation within a small, power-efficient footprint [1,2,3]. These inherent advantages make SDR exceptionally appealing for modern space missions, particularly for the burgeoning small satellite constellations (e.g., CubeSats and MicroSats) [4,5]. These modern satellites increasingly rely on Commercial-Off-The-Shelf (COTS) components to meet demanding constraints on size, weight, power, and cost (SWaP-C) [6]. Among commercially available COTS SDR transceivers, the Analog Devices AD936x family (e.g., AD9361, AD9363, AD9364) stands out as a highly integrated, wideband RF Agile Transceiver [7]. Its compact footprint, low power consumption, extensive configurable features, and impressive RF performance make it an attractive candidate for advanced satellite communication payloads [8,9].

The space environment is characterized by harsh radiation, including energetic protons and heavy ions, whose effects vary with orbital altitude and inclination. A primary concern for integrated circuits is Single Event Effects (SEEs). However, COTS devices like the AD936x are not radiation-hardened, and their highly configurable register map makes them particularly susceptible to Single Event Upsets (SEUs) in the space radiation environment. Extensive radiation testing has confirmed that while the AD9361 is remarkably robust against Total Ionizing Dose (TID) and immune to destructive Single Event Latch-up (SEL) [10,11,12], its primary vulnerability lies in its susceptibility to non-destructive SEUs in its configuration registers. An SEU occurs when an energetic particle strike flips the state of a memory bit, critically including the configuration registers that govern the device’s operation. The goal of a fault-tolerant system is to detect and correct errors caused by faults to prevent system failures. For the highly configurable AD936x, an SEU in a vital control register, which affects frequency synthesis, gain control, or filter characteristics, can lead to catastrophic communication failure [13,14]. While the most robust approach to ensuring reliability is through radiation-hardened (rad-hard) components, which employ circuit-level Radiation-Hardening-by-Design (RHBD) techniques such as Triple-Node Upset (TNU) tolerant latches [15] and Quadruple-Node Upset (QNU) tolerant designs [16], or system-level Triple Modular Redundancy (TMR) [17], these approaches are often unsuitable for modern small satellite missions. Rad-hard chips are prohibitively expensive, suffer from long lead times, and often lag in performance, while TMR imposes a significant overhead in terms of resources, power, and complexity. Therefore, Various Fault Detection, Isolation, and Recovery (FDIR) strategies have been explored for aerospace systems utilizing COTS components. These approaches can be broadly categorized into hardware-centric and software-centric solutions.

Hardware-centric FDIR, as demonstrated by Hörmer et al. for the PRETTY CubeSat mission, often involves a multi-layered power supply monitoring and protection architecture [18]. This approach uses dedicated supervisory ICs and power bus switches to detect overcurrent or undervoltage situations, thereby providing robust protection against destructive events like Single Event Latch-up (SEL). While highly effective for power system integrity, this method primarily addresses hardware failures and does not directly mitigate the more frequent, non-destructive Single Event Upsets (SEUs) in the configuration registers of complex digital components like an SDR transceiver.

For software-based FDIR strategies, a common technique is periodic register scrubbing, where a known-good “golden copy” of the configuration is rewritten to the device to correct bit-flips [19,20,21,22,23]. However, this approach is often insufficient for a stateful device like the AD936x. Due to its intricate internal state machines, overwriting a corrupted register does not guarantee that the device will correctly reload and apply this new value, meaning that many critical changes require specific recalibration procedures to take effect. Performing a full device reset and reconfiguration upon any error is robust but operationally costly. This process can cause an unacceptable interruption in the communication link, compromising the mission’s requirement for continuous operation.

This work proposed a software-defined fault-tolerance framework tailored to the specific characteristics of the AD936x, enhancing the operational reliability of the COTS AD936x RF transceiver in radiation-prone aerospace environments. Our approach is built upon three core techniques: Proactive Register Scrubbing, Real-time Health Monitoring, and Autonomous Fault Recovery. This methodology integrates preventive maintenance (differentiated register scrubbing) with responsive repair (monitoring and recovery), ensuring continuous and robust operation. The core contributions are: (1) A detailed fault classification and a tailored, multi-layered mitigation strategy that applies different handling techniques (e.g., standard scrubbing, masked inspection, procedural refresh) based on register criticality. (2) The design and validation of a low-overhead, purely software-based FDIR system that provides comprehensive protection against the dominant SEE-induced failure modes of the AD936x. (3) A quantitative demonstration of the framework’s effectiveness, showing rapid, deterministic recovery from a wide range of simulated faults with minimal impact on host processor resources. While the specific implementation details and register mapping are unique to the AD936x, the underlying methodology provides a scalable and adaptable blueprint for enhancing the reliability of other highly integrated COTS RF transceivers or complex integrated circuit chips with similar susceptibility profiles.

This paper is organized as follows. Section 2 details the system architecture, analyzes the AD936x’s vulnerabilities based on published radiation test data, and provides an on-orbit event rate analysis. Section 3 presents the proposed software-defined fault-tolerance framework, including its differentiated scrubbing and recovery strategies. Section 4 describes the experimental validation methodology and presents a detailed analysis of the results. Finally, Section 5 concludes the paper and outlines directions for future work.

## 2. System Architecture and Failure Analysis

### 2.1. AD936x Architecture and Its Vulnerability

**Figure 1:**
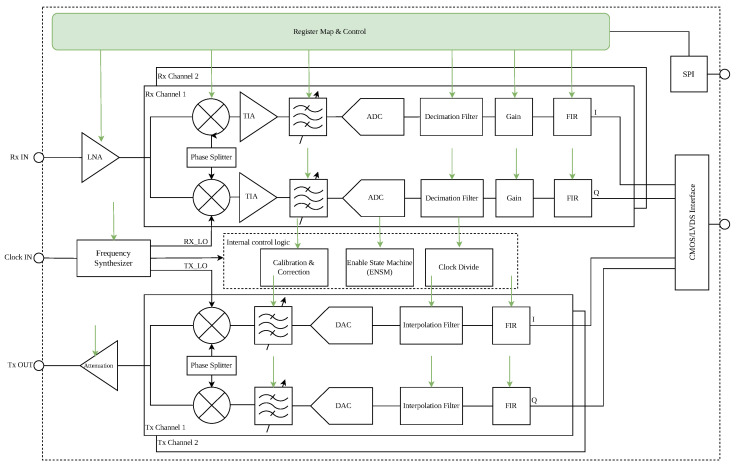
Functional Hardware Architecture of AD936x.

The Analog Devices AD936x is a highly integrated, wideband Radio Frequency (RF) transceiver. As shown in Figure 1, the chip integrates a comprehensive suite of functional blocks, including dual-channel RF front-ends (LNAs, mixers, TIAs), mixed-signal Analog-to-Digital Converters (ADCs) and Digital-to-Analog Converters (DACs), agile frequency synthesizers for local oscillator (LO) generation, and substantial digital signal processing stages such as programmable FIR filters. This complexity is managed through an extensive SPI-accessible register map. The high level of integration makes it an ideal component for SWaP (Size, Weight, and Power)-constrained communication payloads.

The operational behavior of this complex architecture is governed by 1024 internal 8-bit registers. These registers are accessed by a host processor (FPGA or MCU) via a 4-wire Serial Peripheral Interface (SPI), granting fine-grained control over every critical function. While this deep programmability is the AD936x’s primary strength, it is also its greatest vulnerability in a radiation environment. The integrity of the entire communication link is critically dependent on the correctness of the values stored in this vast register space. An SEU may cause: (1) incorrect signal path configuration (e.g., bandwidth mismatch); (2) loss of Local Oscillator (LO) lock; (3) corruption of calibration results; or (4) a device hang requiring a hardware reset.

### 2.2. Failure Modes in the Space Radiation Environment

The deployment of a COTS device like the AD936x in space exposes it to a harsh radiation environment, characterized by energetic particles such as protons and heavy ions, which can induce various SEEs. These include Single Event Upsets (SEUs), Single Event Latch-ups (SELs), Single Event Transients (SETs), and Single Event Functional Interrupts (SEFIs). The most relevant failure modes for the AD936x include:

1. SEU in Configuration Registers: This is the most probable threat. An SEU can flip a single bit, directly altering a critical operational parameter. For example, an upset in the Tx/Rx Synthesizer registers (0x230–0x28F) can cause a loss of frequency lock, while an upset in the FIR filter coefficient registers (0x060–0x065) can distort the signal waveform. Multiple-Bit Upsets (MBUs) can also occur, affecting multiple bits within a single register or adjacent registers, leading to more complex corruption. While Single Event Transients (SETs) are typically associated with combinatorial logic and can manifest as spurious pulses, their direct impact on the AD936x’s register-based control is less prominent compared to SEUs and MBUs.
2. SEU in Control Logic/State Machines: An SEU can also strike the underlying finite state machines (FSMs) that manage the transceiver’s calibrations and operational sequences. This may cause the device to enter a non-responsive or “hung” state, where it no longer responds to SPI commands. Such upsets can also lead to Single Event Functional Interrupts (SEFIs).
3. Single Event Latch-up (SEL): A more destructive SEE, SEL triggers a high-current state that can cause permanent damage if not mitigated by a power cycle. While our framework primarily targets SEUs, the health monitoring component can help flag anomalous conditions that may indicate an SEL, triggering an external supervisory circuit.

### 2.3. Radiation Hardness Characterization

The successful deployment of a COTS device like the AD936x in space missions hinges on a thorough understanding of its response to the radiation environment. Extensive radiation testing of the AD936x, fabricated in 65 nm CMOS technology, has been conducted by multiple parties, providing a clear profile of its strengths and weaknesses. The key findings from heavy ion characterization [10,14] are summarized in Table 1.

A comprehensive heavy ion characterization by Budroweit et al. [10,14] demonstrated the device’s exceptional robustness against destructive failures. Key findings include:

- Total Ionizing Dose (TID): Previous studies referenced in their work confirm the device’s high tolerance to TID, a critical factor for long-duration missions.
- Single Event Latch-up (SEL): The device showed complete immunity to destructive SEL. No events were observed up to an effective Linear Energy Transfer (LET) of 125 MeV·cm^2^/mg, achieved with Xenon ions at a 60° tilt angle. This immunity means that catastrophic, unrecoverable hardware failure from a single particle strike is not a primary design driver.

However, the same studies confirm that the AD9361’s primary vulnerability lies in its susceptibility to non-destructive Single Event Effects (SEEs). The dominant failure mode is Single Event Upsets (SEUs) within its vast configuration register map and internal state machines. Heavy ion testing revealed the following characteristics:

- Device-Level SEU Cross-Section: The device exhibits a clear susceptibility to SEUs, with a saturation cross-section of approximately $2\times 10^{-4}{\mathrm{cm}}^{2}/\mathrm{device}$. Multiple-Bit Upsets (MBUs) were also observed, with a saturation cross-section of around $1.5\times 10^{-5}{\mathrm{cm}}^{2}/\mathrm{device}$.
- SEU-Induced Functional Interrupts (SEFIs): An SEU in a critical register often leads to a SEFI. These were categorized into events recoverable by simple register re-configuration (scrubbing) and more severe events requiring a full device re-initialization (reset)
- Data Path Corruption: Upsets can also manifest as corruption in the transmit (TX) or receive (RX) I/Q data streams, ranging from transient glitches (Soft IQ SEFI) to a complete loss of signal (Hard IQ SEFI), often caused by synthesizer or PLL anomalies.

This specific vulnerability profile—where the device is hardware-robust against destructive events but prone to recoverable, software-visible configuration errors—makes the AD9361 an ideal candidate for the software-defined fault-tolerance framework proposed in this paper. Our approach is designed precisely to manage and autonomously recover from these anticipated SEU-induced events.

### 2.4. On-Orbit Event Rate Analysis and System Implications

The fundamental calculation for the event rate R (in units of upsets/device-second) is given by the integral of the device’s upset cross-section σ(L) over the differential Linear Energy Transfer (LET) spectrum of the particle environment ϕ(L) at the device’s location:

(1) $$\lambda =\int_{L_{min}}^{L_{max}}\phi \left(L\right)\sigma \left(L\right)dL$$

where:

λ: The predicted SEU rate.

$\phi \left(L\right)$: The differential LET flux spectrum (particles/cm^2^·s·MeV·cm^2^/mg) inside the spacecraft, after accounting for shielding. This spectrum is generated by environment models (e.g., AP8/AE8 for trapped protons, CREME96 for GCR) processed by transport codes.

$\sigma \left(L\right)$: The SEU cross-section (cm^2^/device) as a function of the particle’s LET, determined from heavy ion beam testing (as summarized in Table 1).

The discrete experimental data points for σ(L) are typically fitted to a four-parameter Weibull function to provide a continuous curve for the integration in Equation (1). The Weibull function is a standard in the field and is expressed as:

(2) $$\sigma \left(L\right)=\sigma_{\mathrm{sat}}\left\{1-\exp\left[-{\left(\frac{L-L_{\mathrm{th}}}{W}\right)}^{S}\right]\right\}\;\mathrm{for}\;L>L_{\mathrm{th}}$$

where:

$\sigma_{\mathrm{sat}}$: The saturation cross-section, representing the device’s maximum sensitive area (e.g., $∼2\times 10^{-4}\;{\mathrm{cm}}^{2}/\mathrm{device}$ for the AD9361).

$L_{\mathrm{th}}$: The threshold LET, below which the probability of an upset is considered zero.

*W* and *S*: Fitting parameters that define the width and shape of the transition region from the threshold to saturation.

Mean Time Between Failures (MTBF) is the most straightforward metric, representing the average time between two consecutive events.

(3) MTBF=1/λ

Reliability Function R(t) gives the probability that the device will operate without any SEU events for a duration t. It is defined by the exponential reliability law:

(4) $$R\left(t\right)\;=e^{-\lambda t}$$

The Poisson distribution provides a more nuanced view, allowing calculation of the probability of observing exactly k events in a time interval t.

(5) $$P\left(k;t\right)=\left[{\left(\lambda t\right)}^{k}\cdot e^{-\lambda t}\right]/k!$$

where k is the number of events (k = 0, 1, 2, …). A crucial metric for mission risk assessment is the probability of experiencing at least one upset during time t. This is calculated as 1 min the probability of zero upsets (k = 0):

(6) $$P\left(k\geq 1;t\right)=1-P\left(0;t\right)=1-e^{-\lambda t}$$

To quantify the operational risk posed by SEUs, we can analyze the on-orbit event rate predictions provided by Budroweit et al. [10,14]. Their analysis, based on the heavy ion test data and using the OMERE tool, provides a stark picture of the threats in different orbital environments. The results, assuming a standard shielding of 1 g/cm^2^ aluminum, are summarized in Table 2.

Using the worst-case GEO solar flare data from Table 2 (R = 13.3 events/day), we can illustrate with (4) that the probability of the AD9361 surviving just one hour without an upset is

(7) $$R\left(1\mathrm{hour}\right)\;=e^{-0.554\times 1}\approx 0.575$$

or only 57.5%. Using Equation (6), the probability of at least one SEU occurring within that same hour is

(8) 1−0.575=0.425

or 42.5%.

The data presented in Table 2 provides critical insights for system design and mission planning:

1. Nominal vs. Worst-Case Disparity: There is a dramatic, multi-order-of-magnitude difference in SEU rates between nominal conditions and a solar flare event. While the MTBF under nominal conditions might seem acceptable for some short-term missions (several years), the system’s survivability is ultimately dictated by its response to worst-case scenarios.
2. Operational Infeasibility of Manual Recovery: During a solar storm, the AD9361 could experience multiple configuration-corrupting events per day in LEO, and more than a dozen in GEO. The shortest time between failures can shrink to mere hours. In such a scenario, relying on ground-based intervention for detection and recovery is operationally infeasible due to communication latency, link availability, and the sheer frequency of events.

These figures starkly illustrate that an automated, on-board fault tolerance mechanism is not an optional enhancement but an absolute necessity for ensuring mission reliability. During a solar storm, the AD9361 could experience multiple configuration-corrupting events per day, shrinking the time between failures to mere hours. Relying on ground intervention is operationally infeasible due to communication latency and event frequency. The proposed software-defined fault-tolerance framework is therefore a critical enabling technology for deploying the AD936x in reliable, long-duration aerospace applications.

## 3. Proposed Software-Defined Fault-Tolerance Framework

To address the SEU vulnerabilities identified in the device’s radiation characterization, we propose a multi-layered, software-defined fault-tolerance framework. The entire mitigation logic is implemented in firmware, offering a flexible, low-overhead solution that enhances robustness without hardware modifications.

### 3.1. Overall System Design

**Figure 2:**
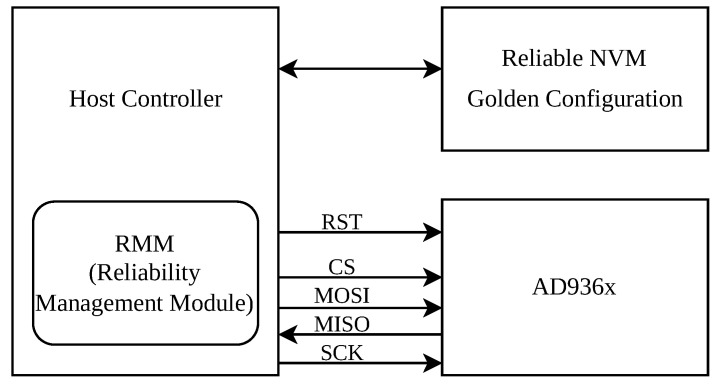
System block diagram illustrating the proposed fault-tolerance framework for the AD936x.

The system architecture, shown in Figure 2, consists of the AD936x transceiver, a host controller (e.g., MCU), and reliable non-volatile memory (NVM).

The Host Controller, typically a radiation-tolerant microcontroller (MCU), serves as the brain of the system, executing the proposed fault-tolerance framework. It is responsible for all mitigation tasks, including managing the AD936x’s configuration, performing register scrubbing, monitoring chip health, and initiating recovery procedures via the SPI bus.

**Figure 3:**
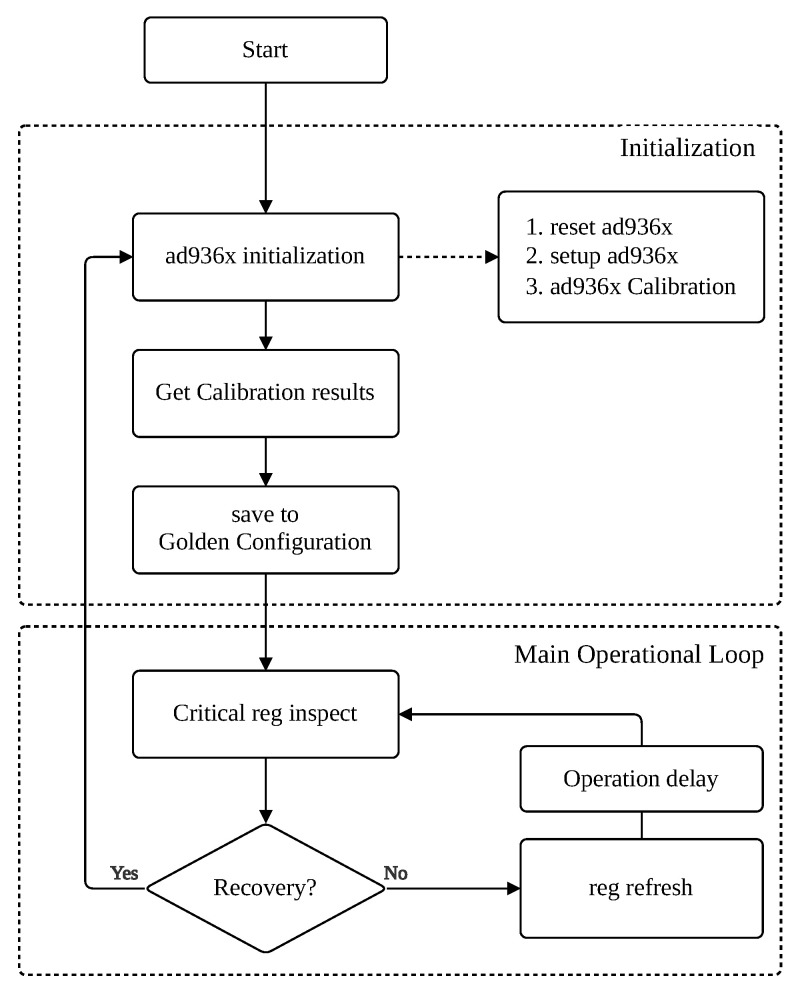
Algorithmic flowchart of the Reliability Management Module (RMM).

The Reliability Management Module (RMM) is the core software component of this framework, implemented within the Host Controller’s firmware. This dedicated module contains the complete logic for the three core techniques: register scrubbing, health monitoring, and autonomous recovery. The overall algorithm is depicted in Figure 3.

Reliable Non-Volatile Memory (NVM) is essential for storing the AD936x’s critical operational parameters. This “Golden Configuration” comprises both a static, pre-defined base configuration and dynamic values, such as one-shot calibration results, that are captured after the initial power-on sequence. This hybrid approach ensures that both fixed settings and device-specific calibration data are preserved. The “Golden Configuration” is stored in a reliable NVM such as a Nor-Flash memory with built-in error detection and correction (e.g., ECC or CRC). This ensures that the reference data used for scrubbing and recovery remains uncorrupted, even in the presence of radiation.

### 3.2. Fault Classification and Mitigation Strategy

To develop an efficient framework, we classify register faults into risk levels, each with a specific mitigation response:

- Risk Level 1 (Critical Failure): An SEU in a bit that can cause a chip-internal reset or lock-up (e.g., in register 0x001). This requires a full chip reset and reconfiguration.
- Risk Level 2 (Major Fault): An SEU corrupts a static configuration register, causing performance degradation. The fault is recoverable by scrubbing the register with its correct “golden” value.
- Risk Level 3 (Minor Fault): An SEU affects registers part of a self-correcting internal loop (e.g., tracking calibrations). The chip’s internal mechanisms automatically correct the value.
- Risk Level 4 (Benign Fault): The SEU occurs in unused or non-critical registers.

This classification forms the basis of our multi-layered mitigation strategy, ensuring the response is proportional to the fault’s severity.

### 3.3. Proactive Mitigation: A Differentiated Scrubbing Approach

**Figure 4:**
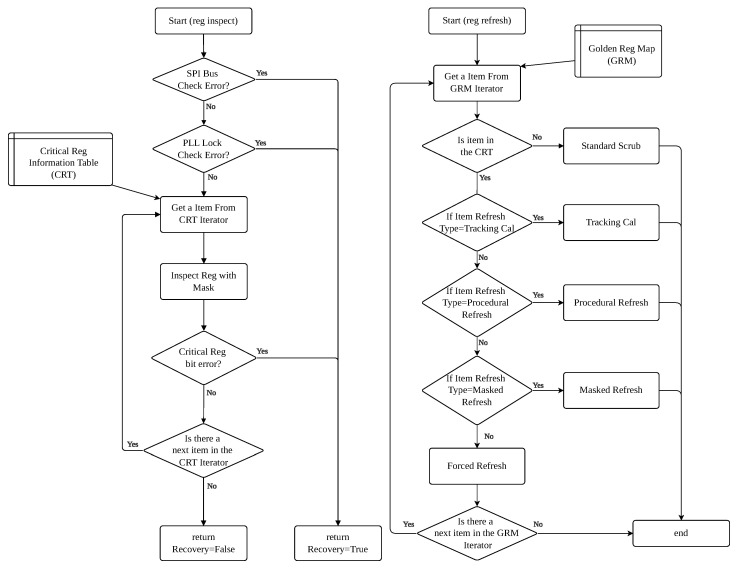
Detailed flowchart of real-time reg inspection and differentiated reg scrubbing.

The core of our proactive strategy is a differentiated register scrubbing methodology, implemented via the “reg refresh” and “reg inspect” functions (see Figure 4), which avoids a naive “one-size-fits-all” approach. The strategy is driven by a special handling table (Critical reg information table) that defines the actions for non-standard registers.

#### 3.3.1. Standard Scrubbing for Static Registers (Risk Level 2)

For the majority of the AD936x registers that hold static configuration values, the RMM periodically writes the value from its “golden copy” (golden reg map) directly to the corresponding AD936x register. This constitutes the baseline defense against most SEUs.

#### 3.3.2. Specialized Handling of Critical and Mixed-Control Registers

Certain registers require specialized handling due to their critical or dynamic nature. This is managed by a dedicated function, which uses bitmasks for targeted operations.

- Masked Scrubbing and Targeted Monitoring (Risk Level 1 & 2): Some registers contain both static configuration bits and critical control or dynamic status bits. For these, we apply masked scrubbing. A prime example is Register 0x001. An SEU in bits [6:5] can trigger an unintended chip reset. Therefore, these bits are designated for inspection only. If an anomaly is detected (*reg inspect*), it triggers a full system reset. The remaining configurable bits ([7] and [4:0]) are safe to be refreshed periodically (*reg refresh*). This dual-mask approach is key to safe scrubbing. The identification of these critical registers and the determination of their specific bit-level functions were derived from a thorough analysis of the AD9361 Register Map Reference Manual [24], corroborated by empirical testing to confirm the functional impact of bit corruptions. Table 3 details this strategy for key registers.
- Procedural Scrubbing for Indirect Registers: Configurations like FIR filter coefficients (e.g., 0x060–0x065, 0x0F0–0x0F6) and AGC gain tables (e.g., 0x130–0x143) are not written to single registers but are loaded through a procedural sequence. Scrubbing these involves periodically re-executing the entire programming procedure provided by the ADI API to refresh their contents from the golden copy.

#### 3.3.3. Management of Calibration Registers

Calibration results are dynamic and require a distinct strategy based on their operational mode.

- Continuous Tracking Calibrations (Risk Level 3): For calibrations supporting continuous tracking, such as Rx Quadrature Correction (0x182), the RMM allows the AD936x’s internal algorithms to operate unimpeded. These registers are deliberately excluded from the scrubbing list, as the internal tracking provides a natural and more efficient SEU mitigation mechanism.
- One-Shot Calibrations with Forced Refresh (Risk Level 2): Calibrations performed once at initialization (e.g., Synthesizer VCO Cal, Tx Quadrature Cal) do not support continuous tracking. For these, a “forced refresh” strategy is employed. After initial calibration, the RMM reads the result, saves it to the golden copy, and sets a “force” bit in the corresponding control register. This allows the RMM to periodically scrub the applied calibration register with the stored golden value, protecting it from SEUs.

The specific mechanism involving the “force” bit and the operational sequences for these one-shot calibrations are based on the procedures outlined in the AD9361 Reference Manual [25]. This ensures that the vital calibration data is protected from SEUs. Table 4 details this approach for key calibrations.

### 3.4. Real-Time Anomaly Detection and Autonomous Recovery

While proactive scrubbing manages latent faults, the RMM’s health monitoring function (reg inspect) serves as the watchdog for immediate and critical failures. Key parameters are continuously checked:

- SPI Communication Integrity: The Product ID register (0x037) is read to ensure the SPI link is functional.
- Synthesizer Lock Status: The LO Lock Status bits (0x247[1] for Rx, 0x287[1] for Tx) are monitored. A persistent unlock indicates a critical failure.
- Critical Bit Integrity: Critical bits in registers like 0x001 and 0x2A6.

The detection of a Level 1 fault—either through targeted bit monitoring or a fatal anomaly like a persistent LO unlock—initiates a full, multi-step recovery procedure: hardware reset, full reconfiguration from the golden copy, re-calibration, and verification, ensuring the system returns to a known-good state.

## 4. Experimental Validation and Results

To validate the efficacy and quantify the performance of the proposed software-defined fault-tolerance framework, which implements a complete Fault Detection, Isolation, and Recovery (FDIR) strategy, we designed and conducted a comprehensive experimental test campaign. The methodology is centered around fault injection, a well-established technique for simulating the effects of SEUs in a controlled laboratory environment without requiring access to a radiation source.

### 4.1. Objectives and Methodology

The primary objectives of the experimental validation were to:

- Verify Effectiveness: Prove that the framework can autonomously detect and successfully correct SEU-induced register corruptions.
- Quantify Performance: Precisely measure the Fault Recovery Time (FRT)—the duration from fault occurrence to the full restoration of system functionality.
- Assess Robustness: Test the system’s response to various fault types, including those in critical and non-critical registers, as well as simulated Multiple-Bit Upsets (MBUs).
- Evaluate Overhead: Analyze the computational overhead imposed by the FDIR software on the host processor during nominal, fault-free operation.

### 4.2. Experimental Setup

**Figure 5:**
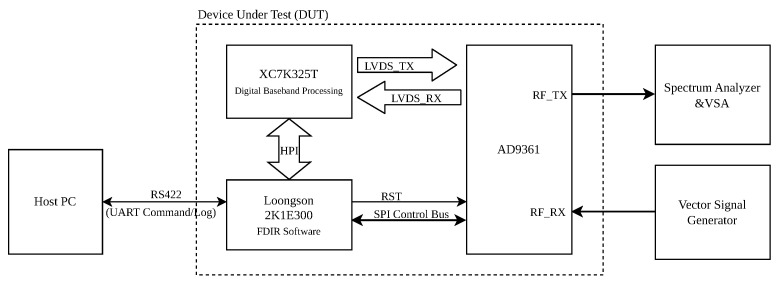
Block diagram of the fault injection experimental setup. The DUT features a Loongson 2K1E300 processor running the FDIR software and a Xilinx Kintex-7 FPGA for baseband processing. The Loongson processor controls the AD9361 via SPI, while the FPGA handles the LVDS data interface.

The testbed, depicted in Figure 5, is comprised of a Device Under Test (DUT), a Host PC for control and monitoring, and standard RF measurement instruments.

The core of the setup is the DUT, which features a heterogeneous computing architecture:

- Control Processor: A Loongson 2K1E300 processor serves as the system controller. It runs a real-time operating system and hosts the FDIR application software proposed in this paper. It is responsible for configuring all components and executing the fault mitigation logic.
- Data Path FPGA: A Xilinx Kintex-7 XC7K325T FPGA implements the high-speed digital baseband processing, including modulation, demodulation, and data formatting. It communicates with the AD9361 via a high-speed LVDS interface for I/Q data transfer and is managed by the Loongson processor through a Host Port Interface (HPI).
- RF Transceiver: The Analog Devices AD9361, which is the target of our FDIR framework. Its configuration registers are directly accessed by the Loongson processor via an SPI control bus.
- Host PC: A standard PC connected to the DUT’s Loongson processor via an RS422 serial interface. It runs a Python (version 3.11.2) automation script to send high-level commands (e.g., “inject fault at address X”), initiate tests, and log telemetry data for post-analysis.
- RF Instruments: A Vector Signal Generator (Rohde & Schwarz GmbH & Co. KG, Munich, Germany) provides a stable, known input signal to the AD9361’s receiver, while a Spectrum Analyzer & Vector Signal Analyzer (Keysight Technologies Inc., Santa Rosa, CA, USA) monitors the transmitter output to verify correct operation and measure recovery performance.

The test procedure for a single run involves: (1) Initializing the DUT to a known “Golden State” with a stable RF output. (2) Sending a command from the Host PC to the Loongson processor to inject a fault by directly writing an erroneous value to a specific AD9361 SPI register. (3) The FDIR software, running on the Loongson, autonomously detects and corrects the fault. (4) The VSA verifies the full restoration of the RF signal, and the Host PC logs the recovery time.

### 4.3. Results and Analysis

A campaign of 10,000 independent fault injections was performed, targeting a diverse set of register locations and fault patterns to thoroughly exercise the fault-tolerance framework. The fault injection campaign was systematically designed to ensure comprehensive testing of all identified fault categories, including single-bit errors in all writable registers, multi-bit errors simulating MBUs, and targeted flips of critical control bits. The results are presented and analyzed below.

#### 4.3.1. Overall Performance Evaluation

Table 5 summarizes the high-level performance metrics of the fault-tolerance framework across the entire test campaign. The results demonstrate the framework’s exceptional reliability and deterministic recovery capability.

Analysis: The most critical finding is the 100% fault coverage rate for the tested scenarios. Across 10,000 simulated SEU and MBU events, designed to cover a diverse range of register types, fault severity levels, and injection patterns, the fault-tolerance framework successfully detected and fully recovered from every single injected fault, restoring the transceiver to its pre-fault “Golden State”. This success rate for our comprehensive test matrix provides strong evidence of the framework’s robustness and its ability to prevent catastrophic system failures originating from register corruption.

The timing performance reveals a mean fault recovery time (FRT) of 749.3 ms. While not instantaneous, this sub-second recovery is sufficiently rapid for the vast majority of aerospace communication protocols, preventing prolonged link outages and ensuring mission continuity. The large standard deviation (721.2 ms) and the wide range between the minimum (164.0 ms) and maximum (4354.0 ms) recovery times indicate the presence of different recovery mechanisms, whose performance is dictated by the nature and severity of the fault. This is explored in detail in the following section.

#### 4.3.2. Detailed Performance by Fault Type

To gain deeper insight into the framework’s behavior, faults were categorized based on the target register’s classification (critical or non-critical) and the nature of the bit corruption. The fault injection experiment was systematically designed to ensure comprehensive testing of all identified fault categories, including single-bit errors in all writable registers, multi-bit errors simulating MBUs, and targeted flips of critical control bits. Table 6 details the performance for each category, showcasing the system’s adaptive response strategy.

Analysis: Table 6 illustrates the framework’s intelligent, tiered recovery strategy.

- Standard Faults (Non-critical registers and non-critical bits within critical registers): This group, comprising 97.71% of the test cases, represents the most common types of SEUs. For these faults, the system’s proactive scrubbing mechanism is the primary recovery vector. The mean FRT for these categories is consistently around 670 ms. This recovery time is primarily dictated by the FDIR task’s execution period, which was set to 2 s in our test configuration. On average, a fault is detected and corrected within half of this period, plus the time required for the SPI transaction, which aligns with the observed ∼670 ms result.
- Severe Faults (Critical bits within critical registers): This category represents the most dangerous, functionality-disrupting events, such as an SEU affecting the PLL lock or triggering an internal reset. For these 229 high-stakes cases, the framework correctly identified the severity and escalated the response to a full, robust device re-initialization. This more comprehensive procedure, while significantly longer with a mean FRT of 3891.2 ms (approx. 3.9 s), guarantees that the system is returned to a known-good, fully calibrated state, eliminating any potential latent errors. This comprehensive procedure involves a hardware reset, a full rewrite of over 1000 register values from the golden configuration, and the execution of multiple time-consuming internal calibration routines (e.g., Synthesizer VCO calibration, Tx Quadrature calibration), which collectively account for the observed recovery time of approximately 4 s.

The inclusion of the 90th percentile metric further reinforces this analysis. For all standard faults, 90% of recoveries were completed within 1165 ms. This provides a reliable upper bound for typical performance. The 4353 ms 90th percentile for severe faults aligns with the maximum observed FRT, confirming that the full re-initialization is a deterministic and time-bounded process.

#### 4.3.3. System Overhead Evaluation

A key requirement for any FDIR system is a minimal computational footprint. Table 7 quantifies the CPU utilization of the FDIR software on the Loongson 2K1E300 processor (Loongson, Beijing, China) during nominal operation.

Analysis: The data in Table 7 confirms that the fault-tolerance framework is exceptionally lightweight. With a net average CPU utilization of only 1.3% on the Loongson processor with the monitoring and scrubbing loop set to a 2-s period. The framework imposes a negligible performance penalty. This low overhead makes it an ideal solution for resource-constrained embedded aerospace systems, where CPU cycles must be preserved for primary application processing. The experimental results collectively provide compelling evidence that the proposed framework is an effective, rapid, robust, and efficient solution for mitigating SEU-induced failures in AD9361-based systems utilizing this specific hardware architecture.

## 5. Conclusions

This paper has presented and experimentally validated a comprehensive, software-defined fault-tolerance framework designed to enhance the reliability of COTS AD936x transceivers in radiation-prone aerospace applications. Our approach moves beyond simple, monolithic scrubbing by integrating three synergistic principles: a highly differentiated, proactive register scrubbing strategy; continuous real-time health monitoring of critical status indicators; and a robust, autonomous fault recovery mechanism.

The cornerstone of our methodology is the classification of registers into risk-aware groups, enabling tailored mitigation techniques such as standard scrubbing, masked inspection, procedural refresh, and a novel “forced refresh” strategy to protect one-shot calibration data. Experimental validation through a 10,000-run fault injection campaign provided compelling quantitative evidence of the framework’s success. A 100% fault coverage rate was achieved for all tested scenarios, demonstrating its ability to handle a vast range of SEU scenarios without failure. The framework exhibited an intelligent, tiered response; non-critical faults were corrected through periodic scrubbing, while severe, functionality-threatening faults correctly triggered a more time-intensive but guaranteed full re-initialization, ensuring a return to a known-good state.

Crucially, the demonstrated fault tolerance is achieved with a negligible computational overhead, consuming only 1.3% of the CPU on average. This efficiency confirms the solution’s suitability for SWaP-C constrained missions. By presenting the AD936x as a representative case study for a broad class of complex, configurable COTS components, this work provides a practical and effective blueprint for enhancing the operational robustness to meet the stringent demands of space environments. While this work focuses on the AD936x as a representative and widely used RF transceiver in small satellite applications, the proposed methodology’s core principles are transferable to other complex COTS integrated circuit chips.

Building upon this framework, future work will proceed in two primary directions. First, we aim to demonstrate the scalability and adaptability of our methodology by expanding the approach to other high-integration RF transceivers, such as the next-generation Analog Devices ADRV900x series. This will involve tailoring the register classification and differentiated scrubbing strategies to a new, more complex architecture. Second, we will explore a hybrid FDIR model that combines our robust software scrubbing with lightweight hardware monitoring. Integrating dedicated, low-power circuits to watch for critical events like synthesizer unlock could further reduce fault detection latency and create an even more resilient solution for future deep-space or long-duration missions.

## Figures and Tables

**Table 1:** Summary of Heavy Ion Induced Single Event Effects on the AD9361 Transceiver.

Effect Type	Category	Test Result	Impact on Device Functionality	MitigationRelevance for This Work
Single Event Latch-up (SEL)	Destructive	Immune (No events up to LET_eff = 125 MeV· ${\mathrm{cm}}^{2}$/mg)	Catastrophic, unrecoverable hardware failure.	Low. Not required due to immunity.
Single Event Upset (SEU)	Non-Destructive	$\sigma_{sat}\approx$ 2 $\times 10^{-4}$ ${\mathrm{cm}}^{2}$/device	Corruption of one or more bits in the configuration register map.	High. The primary target for mitigation.
Multiple-Bit Upset (MBU)	Non-Destructive	$\sigma_{sat}\approx$ 1.5 $\times 10^{-5}$ ${\mathrm{cm}}^{2}$/device	More complex corruption within a single register word.	High. Handled by register scrubbing.
Single Event Functional Interrupt (SEFI)—Register Corruption	Non-Destructive	Caused by SEU/MBU in critical registers.	Loss of functionality (e.g., PLL unlock, incorrect filter settings).	High. Addressed by scrubbing/reset.
SEFI—Data Path Corruption (IQ)	Non-Destructive	Caused by upsets in synthesizers or state machines.	Transient glitches or persistent loss of the TX/RX I/Q data stream.	High. Addressed by reset/re-init.

**Table 2:** On-Orbit SEU Rate Projections for the AD9361 Transceiver (Based on data from Budroweit et al. [10,14]).

Orbit Type	Solar Condition	Predicted SEU Rate (Events/Device/Day)	Mean/Shortest Time Between Failures (MTBF/STBF)
Low Earth Orbit (LEO)	Nominal Space Environment	$3.98^{-4}$	∼2512 days (approx. 6.9 years)
(850 km Sun-Synchronous)	Worst-Case Solar Flare (1 day)	3.06	∼7.8 h
Geostationary Orbit (GEO)	Nominal Space Environment	$1.17\times 10^{-3}$	∼855 days (approx. 2.3 years)
	Worst-Case Solar Flare (1 day)	13.3	∼1.8 h

**Table 3:** Differentiated Mitigation Strategies for Critical Registers.

Register	Function / Group	Implementation Detail
0x000	SPI Comms Mode	Periodically read and verify Product ID. Anomaly triggers a full chip reset.
0x017	Chip State	Periodically inspect register. Anomaly triggers a full chip reset.
0x05E	CH 1 Overflow Status	Periodically inspect bit [7]. Anomaly triggers a full chip reset.
0x247	Rx Synthesizer Status	Inspect bit [1] (LO Lock). Anomaly triggers a full chip reset.
0x287	Tx Synthesizer Status	Inspect bit [1] (LO Lock). Anomaly triggers a full chip reset.
0x001	MCS & Tx Mon Control	Scrub: [7] & [4:0]. Inspect: [6:5]. Anomaly triggers a full chip reset.
0x002–0x003	Channel & Filter Control	Scrub: [5:0]. Inspect: [7:6]. Anomaly triggers a full chip reset.
0x009, 0x010	Clock & Parallel Port Configuration	Scrub: [7:2] & [0]. Inspect: [1]. Anomaly triggers a full chip reset.
0x00A	BBPLL	Scrub: [7:4] & [2:0]. Inspect: [3]. Anomaly triggers a full chip reset.
0x012	Parallel Port Configuration 3	Scrub: [7:6] & [3:0]. Inspect: [5:4]. Anomaly triggers a full chip reset.
0x013	ENSM Mode	Scrub: [7:1]. Inspect: [0]. Anomaly triggers a full chip reset.
0x014	ENSM Config 1	Scrub: [7:3] & [1:0]. Inspect: [2]. Anomaly triggers a full chip reset.
0x06E	TPM Mode Enable	Scrub: [7:5] & [3:0]. Inspect: [4]. Anomaly triggers a full chip reset.
0x23A,0x27A	Rx/Tx VCO Output	Scrub: [7] & [5:0]. Inspect: [6]. Anomaly triggers a full chip reset.
0x2A6	Master Bias Config	Scrub: [6:0]. Inspect: [7]. Anomaly triggers a full chip reset.
0x060–0x065	Tx FIR Filter Coefficients	Periodically re-execute the full programming sequence via API.
0x0F0–0x0F6	Rx FIR Filter Coefficients	Periodically re-execute the full programming sequence via API.
0x130–0x143	AGC Gain/Mixer Tables	Periodically re-execute the full programming sequence via API.
0x25A–0x25F	Rx Fast Lock Profiles	Periodically re-execute the full programming sequence via API.
0x29A–0x29F	Tx Fast Lock Profiles	Periodically re-execute the full programming sequence via API.
See Table 4	One-Shot Calibrations	Set “Force” mode after initial calibration, then periodically scrub with saved golden value.
0x182	Rx Quadrature Correction	Excluded from scrubbing list to allow the chip’s internal algorithms to operate unimpeded.

**Table 4:** Forced-Refresh Strategy for One-Shot Calibrations.

Calibration	Force Enable Register/Bit	Result Register(s)	Applied Register(s)	Refresh Method Summary
RX RF Synthesizer Charge Pump	0x23D[3]	0x244[3:0]	0x241[3:0]	Read result after init, set Force mode, periodically scrub 0x241.
RX RF Synthesizer VCO	0x238[1]	0x237, 0x238[0]	0x237, 0x238[0]	Read result after init, set Force mode, periodically scrub 0x237 & 0x238.
RX Synthesizer ALC	0x236[7]	0x236[6:0]	0x236[6:0]	Read result after init, set Force mode, periodically scrub 0x236.
TX RF Synthesizer Charge Pump	0x27D[3]	0x284[3:0]	0x281[3:0]	Read result after init, set Force mode, periodically scrub 0x281.
TX RF Synthesizer VCO	0x278[1]	0x277, 0x278[0]	0x277, 0x278[0]	Read result after init, set Force mode, periodically scrub 0x277 & 0x278.
TX Synthesizer ALC	0x276[7]	0x276[6:0]	0x276[6:0]	Read result after init, set Force mode, periodically scrub 0x276.
TX Quadrature Calibration	0x09F	0x08E–0x09D	0x08E–0x09D	Read results after init, set Force mode, periodically scrub 0x08E-0x09D.

**Table 5:** Overall Fault-Tolerance Framework Performance Summary.

Metric	Value	Description
Total Faults Injected	10,000	The total number of SEU/MBU simulations performed.
Faults Successfully Detected	10,000	Number of injected faults that were correctly identified by the FDIR software.
Faults Successfully Recovered	10,000	Number of detected faults where the system returned to the “Golden State”.
Fault Coverage Rate	100%	The percentage of injected faults that were successfully recovered.
Mean Fault Recovery Time (FRT)	749.3 ms	The average time from fault injection to full functional recovery.
Minimum FRT	164.0 ms	The best-case recovery time, typically for a non-critical register scrub.
Maximum FRT	4354.0 ms	The worst-case recovery time, involving a full device re-initialization.
FRT Standard Deviation	721.2 ms	Indicates the variability in recovery time, dominated by the type of fault.

**Table 6:** Detailed Fault Recovery Performance by Fault Type.

Fault Injection Category	# Injections	Coverage (%)	Mean FRT (ms)	Std. Dev. (ms)	90th Percentile (ms)
Non-critical (single-bit)	1144	100%	674.2	523.2	1165
Non-critical (multi-bit)	6856	100%	678.7	550.0	1165
Critical (non-critical bit)	1771	100%	664.7	500.0	1165
Critical (critical bit)	229	100%	3891.2	499.0	4353
Total/Weighted Avg.	10,000	100%	749.3	721.1	1165

**Table 7:** FDIR Software System Overhead Analysis on the Loongson 2K1E300.

System State	Mean CPU (%)	Peak CPU (%)	Description
Baseline (OS Idle)	0.5%	1.2%	Inherent load of the embedded RTOS.
FDIR Active (Scrub @ 2 s)	1.8%	4.5%	Load with FDIR actively performing checks.
Net FDIR Overhead	1.3%	3.3%	Marginal CPU resources consumed by the framework.

## Data Availability

The raw data supporting the conclusions of this article will be made available by the authors on request.
